# Simulation-Based Curriculum for Medical Undergraduates in Non-technical Skills

**DOI:** 10.7759/cureus.86020

**Published:** 2025-06-14

**Authors:** Manpreet Kaur, Rashmi Ramachandran, Abhishek Nagarajappa, Devalina Goswami, Thilaka Muthiah, Bharat Yalla, Ambuj Roy

**Affiliations:** 1 Anesthesiology and Perioperative Medicine, Penn State Health Milton S. Hershey Medical Center, Hershey, USA; 2 Anesthesiology, All India Institute of Medical Sciences, New Delhi, New Delhi, IND; 3 Anesthesiology, Pain Medicine and Critical Care, All India Institute of Medical Sciences, New Delhi, New Delhi, IND; 4 Anesthesiology, Apollo Simulation Center, Chennai, IND; 5 Anesthesiology, Apollo Hospitals, Chennai, IND; 6 Anesthesiology, Apollo Institute of Medical Sciences and Research, Chittoor, IND; 7 Cardiology, All India Institute of Medical Sciences, New Delhi, New Delhi, IND

**Keywords:** curriculum planning, kern's six steps, medical center, simulation in medical education, teaching and training medical and nursing students and faculty

## Abstract

Introduction

Teaching and training non-technical skills (NTS) at an early stage of education can lay a strong foundation at the root level and hence ensure organizational safety culture. This study aims to describe the process through which the undergraduate curriculum was created and modified based on the needs of the students following Kern's model.

Materials and methods

After institutional ethical approval, a curriculum design using Kern's model was used to teach NTS to undergraduates (UGs) who have completed the third year of medical education, a pilot curriculum design combined didactic lectures with practical simulation sessions and debriefings. The revised curriculum was formulated based on the adjustments made based on students' needs. Implementation of the curriculum on 38 students, in a group of eight to 10, was done.

Results

A general needs assessment was established after evaluating 102 responses. Forty-eight percent opined that NTS was extremely important for patient care. About 80.4% of students recommended simulation-based teaching, and 83.3% recommended bedside problem solving. Based on the curriculum gap, crisis or non-crisis scenarios were used to train on NTS. Both lecture and simulation scenarios were used for educational strategies and program implementation. Multiple changes were incorporated into the pilot curriculum based on the roadblocks. The curriculum was implemented with 38 students, and changes in students’ behavior were assessed along with focused group discussions. All the students found the curriculum useful, and 53.8% supported that the lecture was also a very important part of the teaching modality. A revised curriculum was formulated based on students' needs.

Conclusion

Simulation and lecture-based curriculum for NTS incorporation in formal education programs of undergraduates using the Kern's six-step model was found to be well accepted by UG students.

## Introduction

Technical skills are commonly taught at various levels of medical education. However, there is a lack of teaching and training in non-technical skills (NTS), essential for well-coordinated teamwork, reduced critical events, and effective crisis resource management. Non-technical skills are 'cognitive, social, and personal resource skills,' complementing technical skills and contributing to safe and efficient task performance [1]. Absence or ignorance of non-technical skills increases error chances and leads to an increase in adverse events. Good non-technical skills, such as vigilance, anticipation, clear communication, situational awareness, decision-making, teamwork, and leadership, decrease accidents and support technical skills [2].

Presently, there is an urgent need to develop structured training in non-technical skills for all clinicians. Identifying, training, and evaluating non-technical skills at the undergraduate level can lay a firm foundation at the root level, ensuring organizational safety culture. Simulation is an excellent tool for training and assessing non-technical skills [3,4].

Previous studies have described non-technical skills in a specific cohort of clinicians, for example, anesthetists' non-technical skills for anesthesiologists, non-technical skills for surgeons, and scrub practitioners' list of intra-operative non-technical skills for scrub nurses [5,6]. The present study was designed to incorporate key non-technical skills into the curriculum for undergraduate students. Our curriculum used anesthetists' non-technical skills and a few other common non-technical skills purportedly relevant to undergraduate students.

We aimed to create a seventh semester (undergraduate who has completed third year) curriculum (lecture and simulation-based) that improves the existing curriculum by identifying and introducing some essential non-technical skills that undergraduate students need to be trained in to handle critical and non-critical clinical situations. We also aimed to evaluate the usefulness of such a program using student feedback.

## Materials and methods

The study was conducted in the Skills, E-learning, and Telemedicine Facility, All India Institute of Medical Sciences, New Delhi, India, after Institutional Ethics Committee approval (IEC-342/08.05.2020, RP-25/2020). The inclusion criterion was undergraduate students in the seventh semester.

Study model

The program's curriculum design combined didactic lectures, practical simulation sessions, and debriefings. The simulation scenarios were designed to cover critical, commonly faced clinical situations (Table 1).

**Table 1:** Scenarios for crisis or non-crisis situations

Anaphylaxis
Hemorrhagic shock
Septic shock
Respiratory distress
Myocardial infarction
Cardiac arrest
Unconscious patient
Stroke
Opioid overdose
Hypothermia
Medication error

We developed a pilot curriculum based on the Kern's six-step model of Curriculum Development for Medical Education [7]. After reviewing the curriculum outcomes, adjustments were made based on optimal resource utilization and impact on students' needs. The six steps included (1) problem identification and general needs assessment, (2) targeted needs assessment, (3) goals and objectives, (4) educational strategies, (5) implementation, and (6) evaluation and feedback [7,8].

Problem Identification and General Needs Assessment (Kern's Step 1)

Non-technical skills are essential as they can increase patient safety, but these skills are rarely taught to undergraduates at our Institute. We conducted needs assessments by surveying the seventh-semester students using Google Forms (Google LLC, Mountain View, United States).

Targeted Needs Assessment (Kern's Step 2)

Students in the seventh semester are ideal as they have learned many technical skills but need to learn to work as a team. They are also posted to start working with patients in clinical areas, so their need for learning non-technical skills is maximal.

Goals and Objectives (Kern's Step 3)

We developed goals and objectives for teaching and training undergraduates (UGs) based on target areas identified by need assessment. The primary goal was to create a lecture and simulation-based curriculum to train UG students in practicing non-technical skills during clinical crises.

Educational Strategies Adopted Included Lecture and Simulation Scenarios (Kern's Step 4)

Educational strategy: 1. Lecture: The faculty instructor undertook a 15- to 20-minute seminar targeting sensitizing students to teamwork-supporting behavior and attitudes, situation awareness, team coordination, and feedback rules. The students were familiarized with possible obstacles to communication and teamwork. A video was played on the crisis, and potential positive and negative targeted behaviors were highlighted. The list of non-technical skills included situational awareness, decision-making and prioritization, teamwork and coordination, leadership, stress management, and fatigue management. The lecture/presentation included multiple videos on troubleshooting appropriate and inappropriate behaviors for nontechnical skills. This lecture was reviewed and modified to address the needs of the UGs. 2. Simulation scenarios: We planned the simulation sessions based on the UGs' needs. The simulation resources needed included a medium-fidelity Advanced Cardiac Life Support (ACLS) manikin (Artificial Manual Breathing Unit (AMBU) Advanced Cardiac Care System, Ambu, Copenhagen, Denmark), an airway simulator, a simulator operation manager, and two to three local faculty members as simulation-based educators. We created at least 10 simulation scenarios based on the set-up of a ward, emergency room (ER), labor room, or operation room (OR). Scenarios were designed to simulate crisis or non-crisis situations, as mentioned in Table 1.

A whole-body patient manikin or simulator was used for simulation scenarios. All medications and equipment required to manage the simulated patient were provided. The scenarios were written in advance to cover the critical, commonly encountered medical scenarios for the seventh-semester trainees. They were conducted using a medium-fidelity patient simulator (Adult Advanced Cardiac Life Support with integrated skill reporter: Artificial Manual Breathing Unit (AMBU) Advanced Cardiac Care System, Ambu, Copenhagen, Denmark). One anesthesiologist recorded the scenarios using an audio-visual recording system. We ran each scenario for 40 minutes (pre-briefing, running scenario, and debriefing).

Implementation (Kern's Step 5)

Each curriculum session was chosen from eight to 10 students in the seventh semester. We conducted five sessions with a total of 38 students. All the students were given a questionnaire on non-technical skills, which was followed by a didactic lecture on non-technical skills. One to two simulation scenarios were run for teaching and training, followed by an NTS assessment and questionnaire.

Evaluation and Feedback (Kern's Step 6)

We assessed the students both qualitatively and quantitatively.

Assessment of skills after the simulation scenario: Two trainers, who were expert faculty on non-technical skill training courses, were recruited to assess NTS. Two trainers obtained a definitive rating for each non-technical skill demonstrated in the scenario, as mentioned in Table 2.

**Table 2:** Non-technical skills to be introduced in MUTiNTS MUTiNTS: medical undergraduate training in non-technical skills.

Situational Awareness	Decision Making	Communication Skills	Team Working	Leadership	Stress Management	Fatigue Management
Gathering information	Defining the problem	Giving information clearly and concisely	Supporting others	Using authority and assertiveness	Identifying symptoms of stress	Identifying symptoms of fatigue
Recognizing and understanding	Identifying the options	Including context and intent	Solving conflicts	Maintaining standards	Recognizing the effects of stress	Recognizing the effects of fatigue
Anticipating	Balancing risks and selecting options	Receiving information	Exchanging information	Planning and prioritizing	Implementing coping strategies	Implementing coping strategies
	Reassessing outcomes	Identifying and tackling barriers	Coordinating activities	Managing workload and resources		

They rated the scenarios individually and then discussed their ratings to produce an agreed-upon reference rating. Active feedback was given to the students while they practiced.

Quantitative component: During the session, the students were asked to complete a feedback form regarding the usefulness of the lecture alone and the lecture with simulation. A Likert scale-based evaluation form was prepared for feedback. Objective Structured Clinical Examination (OSCE) was done at the end. All the assessments were done by faculty members involved with the Skills, E-learning, and Telemedicine (SET) Facility.

Qualitative component: Focused group discussions (FGDs) were conducted to gain insight into the learners' perceptions of NTS's usefulness. A homogeneous group of five to seven trainees was selected and invited to participate in these FGDs.

## Results

Problem identification and general needs assessment (Kern's step 1) and targeted needs assessment (Kern's step 2)

**Figure 1:**
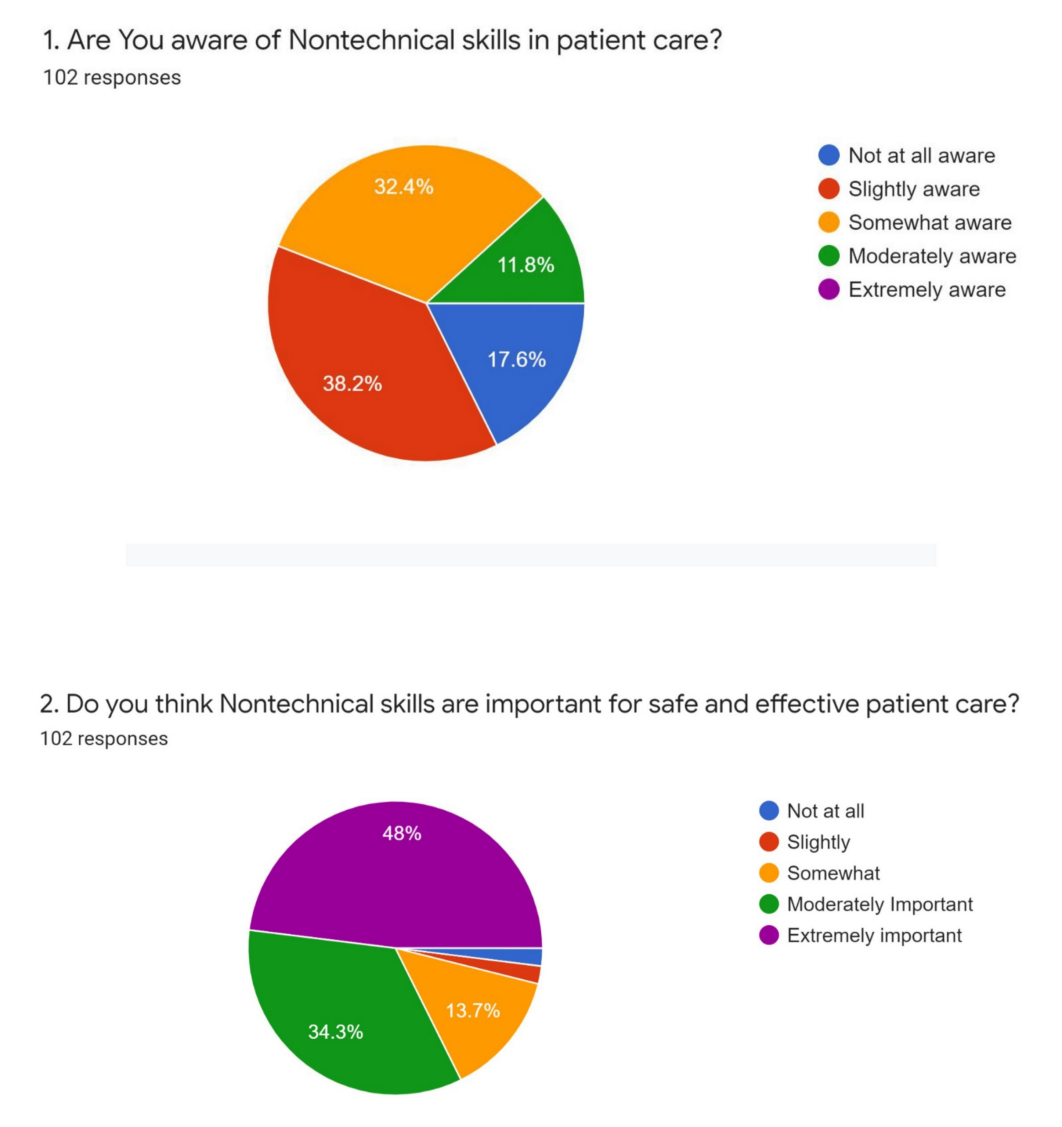
Pie diagram depicting the percentage of student's awareness regarding the importance of non-technical skills in patient care

We evaluated 102 responses using Google Forms, with each student being allowed a single response for the first two questions and multiple for the last question. We found that 17.6% of students were not at all aware, 38.2% were slightly aware, 32.4% were somewhat aware, 11.8% were moderately aware, but none was highly aware of the non-technical skills in patient care. Despite the lack of awareness, 48% opined that NTS was essential for patient care (Figure 1). Forty-eight percent of students felt that NTS were extremely important, 34.3% of students opined that they were moderately important and 13.7% of students thought they were somewhat important (Figure 1).

**Figure 2:**
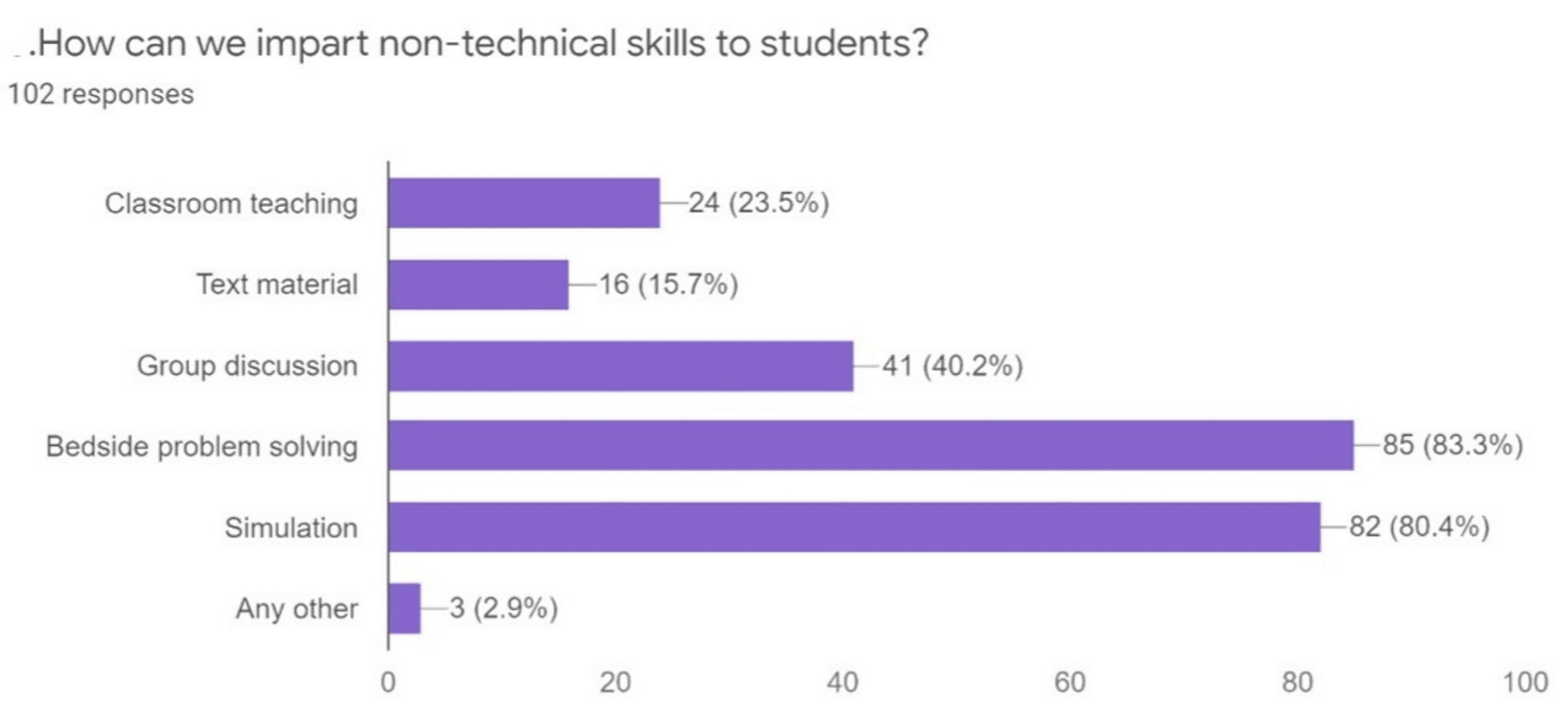
Methods to impart non-technical skills to the students

About 80.4% of students recommended simulation-based teaching, and 83.3% recommended bedside problem-solving as a mode of education (Figure 2).

We discussed the results with the educational leader among faculty, and necessary modifications were made. Findings were supported later by the focused group discussions (FGDs) during the individual group sessions.

Goals and objectives (Kern's step 3)

Goals and objectives were developed based on the curriculum gap. Objectives for training in the seventh semester were to train on non-technical skills in scenarios for crisis or non-crisis situations like anaphylaxis, hemorrhagic shock, septic shock, respiratory distress, myocardial infarction, opioid overdosage, and care of unconscious patients.

Educational strategies and program implementation (Kern's step 4/5)

Both lecture and simulation scenarios were run to familiarize them with non-technical skills, and appropriate interventions were taken to modify the pilot curriculum. Multiple changes were incorporated into the pilot curriculum based on the road blockers, which have been summarized in Table 3.

**Table 3:** Barriers to the implementation of the pilot curriculum and revisions NTS: non-technical skills.

Barrier	Pilot Curriculum	Revised Curriculum
Technical knowledge	It was absent	Technical knowledge about a few commonly encountered scenarios like bradycardia, hypotension, hypoglycemia, hypothermia, and hyperthermia by provision of handouts one day prior
Complicated scenarios	Anaphylaxis, hemorrhagic shock, septic shock, respiratory distress, myocardial infarction, opioid overdosage, care of unconscious patient	Replaced with more commonly encountered scenarios: Hypoxia, postoperative pain, bradycardia, bleeding, reaction to blood transfusion, hypoglycemia, hypotension, hyperthermia, hypothermia
Duration of lecture and simulated session	1 hr and 1 hr, respectively	30 min and two scenarios (30-40 min each)
Personnel	Needed three faculty coordinators (two simulation scenarios, one technical airway training)	Needed two faculty coordinators (or one faculty and one senior resident by incorporating the concept of training the trainers)
Time	Variable clinical schedules of residents and faculty training	Prefixing the training time at the end of the posting and before the ward leaving examination
NTS evaluation	Stress management and fatigue management were difficult to evaluate, hence later excluded	Situational awareness, decision-making and prioritization, teamwork, and coordination, leadership were evaluated

We phased in a 'train the trainer' concept, where senior residents taught the basic technical airway education instead of faculty trainers.

Evaluation and feedback (Kern's step 6)

**Figure 3:**
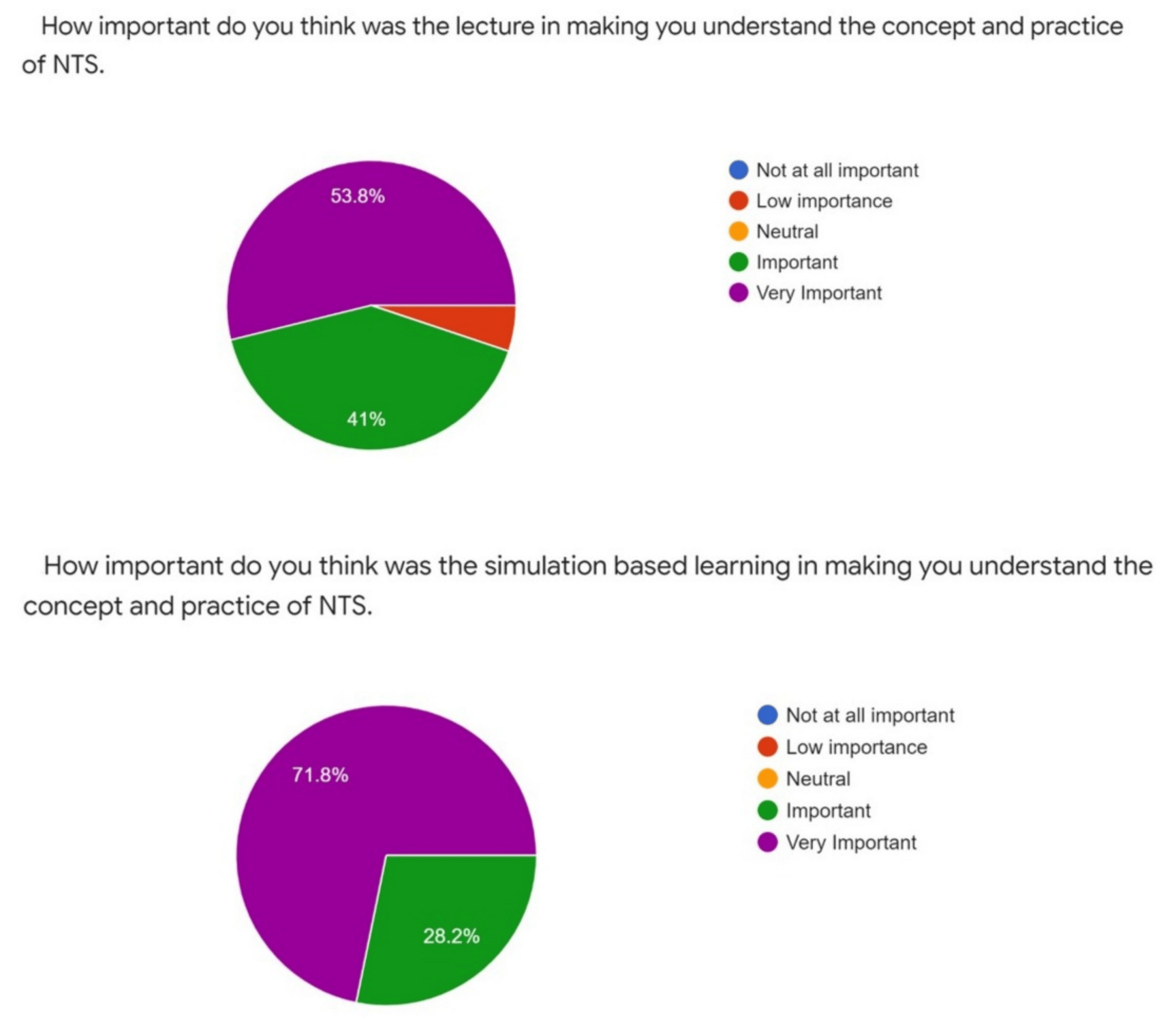
Pie diagrams depicting student-centered importance of lecture versus simulation for understanding non-technical skills NTS: non-technical skills.

After implementing the curriculum on 38 students, changes in students' behavior were assessed by simulation-based scenarios. Faculty feedback was taken regarding the whole program (i.e., utility, usability, problems, and roadblocks in implementation). Then, the entire curriculum was re-assessed, necessary changes were made, and a revised curriculum was made. All the students found the curriculum helpful; 53.8% supported that lectures were also an essential part of the teaching modality for introducing non-technical skills, and none considered them an important part. About 71.8% of students considered simulation a critical part of teaching NTS, and none thought it was nonessential (Figure 3).

In FGDs, multiple students opined that technical skills should also be included along with NTS so that close liaison can result in better training.

Revised Curriculum

A revised curriculum was formulated based on multiple roadblocks and student feedback. A pre-handout was given to the students, which included technical knowledge of managing more commonly encountered scenarios like hypoxia, postoperative pain, bradycardia, bleeding, reaction to blood transfusion, hypoglycemia, hypotension, hyperthermia, and hypothermia. We replaced a bit complex scenarios (anaphylaxis, hemorrhagic shock, septic shock, respiratory distress, myocardial infarction, opioid overdosage, care of unconscious patient) with more commonly encountered scenarios.

## Discussion

Structured training in NTS for medical undergraduates can lay a strong foundation for future medical professionals. NTS has been incorporated into Cardiac Life Support teaching, including Advanced Cardiac Life Support, Paediatric Advanced Life Support, and Neonatal Resuscitation Program [9]. There are few studies about nursing training, but limited studies on NTS training in UG education and training [10-12]. Since there is evidence of favorable outcomes from teaching NTS, we recommend the incorporation of NTS in UG training [13].

We used Kern's six-step approach to develop the UG NTS curriculum [8]. We used Kern's model as it adequately addresses the specific learner needs and is dynamic in that steps 1 and 2 can be done concurrently. It also has the provision of evolving the curriculum. For the implementation of the curriculum, a few aspects to be considered include that these NTS must be complemented with technical knowledge about commonly encountered situations like hypoxia, postoperative pain, bradycardia, bleeding, reaction to blood transfusion, hypoglycemia, hypotension, hyperthermia, and hypothermia. The pilot curriculum was modified based on the learners' needs, including common scenarios, according to the learners' needs at the UG level.

Stepwise evaluation of the Kern's model depicted that UGs need assessment early, i.e., before starting the clinical posting. This is necessary as the highest functioning teams help establish a safety culture. NTS training covers many aspects of team building and training that may significantly impact the care of emergency and critically ill patients [14,15]. Team dynamics is best learned through hands-on practice in tiny, supervised groups. Course-based training enables uniform NTS training among learners, which can be implemented later in real-life scenarios. This training focuses on close cohesion between task completion and team cohesion.

Implementing our pilot curriculum was feasible and well accepted by the learners using combined lecture and simulation as training. The length of the comprehensive curriculum course and teaching techniques (lecture and simulation) were highly appreciated by the learner and appeared adequate for teaching the selected NTS. Debriefing at the end of the session is highly beneficial for the learner to improve upon the NTS and create effective teams. Problems like fixation errors, communication gaps, and overtaxing any individual can be reduced by teaching NTS at an early stage of learning, i.e., UGs.

The learners' experience with the pilot curriculum helped formulate a revised curriculum based on multiple roadblocks. We introduced technical knowledge about commonly encountered scenarios, used simpler scenarios, increased the duration of the simulated session, and removed difficult-to-assess NTS (stress, fatigue). The timing of the session was predetermined by conducting it just before the ward left.

The present study is novel as it proposes to include commonly encountered scenarios in the emergency room (ER) and post-anesthesia care unit in a simulated environment, focusing mainly on NTS training for UG students. However, it does not replace actual clinical experiences; instead, it prepares the UG for field training using a simulated environment. After NTS training, learners perceived improvement in their clinical postings, as expressed in the FGDs.

A few limitations were that the sample size was minimal (38 students) and that it took us eight months to implement the program. The program could become resource-intensive in terms of time and effort, as training 120 UG students each semester can be challenging. However, after gaining experience, this time can be reduced.

Future studies should research in detail how undergraduate NTS training can translate to better patient care. Incorporating NTS into the curriculum can result in excellent team dynamics and be a natural routine in daily clinical practice. We suggest annual curriculum renewal using regular small FGDs.

## Conclusions

The incorporation of simulation as a dynamic learning approach for developing non-technical skills (NTS) within formal undergraduate education programs has proven to be exceptionally well-received by students. By employing Kern's six-step model, this innovative method captivated undergraduate students, providing them with engaging, hands-on experiences that enriched their understanding and application of essential skills in real-world scenarios.
